# How Personality Types Shape the Link Between Nursing Work Environment and Subjective Well‐Being

**DOI:** 10.1155/jonm/1147669

**Published:** 2026-11-04

**Authors:** Mingxuan Wang, Xianjing Hu, Peitao Li, Heli Zhang, Rongmei Geng, Baohua Li

**Affiliations:** ^1^ Peking University Third Hospital Beijing China puh3.net.cn

**Keywords:** latent profile analysis, moderating effect, nurses’ subjective well-being, nursing work environment, personality types

## Abstract

**Objectives:**

Guided by the Cognitive‐Affective Personality System theory, this study aimed to identify Big Five personality profiles among clinical nurses and test the hypotheses that favorable nursing work‐environment dimensions would be positively associated with subjective well‐being and that these associations would differ across personality profiles.

**Methods:**

This cross‐sectional study included 1186 nurses from a tertiary hospital in Beijing. Participants completed the General Well‐Being Schedule, the Chinese Big Five Personality Inventory‐15, and the Nursing Work Environment Scale. Latent profile analysis identified personality profiles, and hierarchical regression examined moderation effects. Bonferroni correction was applied across seven moderation models.

**Results:**

Three personality profiles were identified: resilient (43.17%), cautious (50.08%), and independent (6.75%). All seven nursing work‐environment dimensions were positively associated with subjective well‐being. After Bonferroni correction, personality profile significantly moderated the associations of recognition climate (Δ*R* ^2^ = 0.006, *p* = 0.002) and professional autonomy (Δ*R* ^2^ = 0.005, *p* = 0.006) with subjective well‐being. For recognition climate, the positive association was weaker among cautious nurses than among resilient nurses (*β* = −0.140, *p* < 0.001), whereas independent nurses did not differ significantly from resilient nurses. A similar pattern was observed for professional autonomy: the positive association was weaker among cautious nurses than among resilient nurses (*β* = −0.121, *p* < 0.01), whereas independent nurses did not differ significantly from resilient nurses. No significant moderation was found for the other five work‐environment dimensions.

**Conclusion:**

Personality profiles showed a selective and small moderating role, with significant moderation involving recognition climate and professional autonomy. In both models, the positive association with subjective well‐being was weaker among cautious nurses than among resilient nurses. Broad improvement of the nursing work environment should remain the primary management priority, while personality‐informed support may be used cautiously as a supporting strategy.

**Implications for Nursing Management:**

Nursing managers should prioritize broad improvements in the nursing work environment, provide recognition that is specific, timely, supportive, and focused on professional contributions rather than interpersonal comparison, and support professional autonomy with clear decision boundaries, procedural guidance, and accessible supervisory support. Personality‐informed support may be considered cautiously as a supporting strategy.

## 1. Introduction

Nurses’ subjective well‐being (SWB) is not only an individual mental health issue but also an important workforce concern because it is associated with work engagement, emotional exhaustion, burnout, and turnover intention [1–3]. Hospital nurses are frequently exposed to heavy workloads, emotional demands, work–family conflict, and stressful clinical situations, which may undermine their well‐being and increase emotional exhaustion [1, 4, 5]. Lower well‐being and greater burnout may threaten workforce stability. Identifying modifiable organizational factors associated with nurses’ SWB is therefore important for maintaining a healthy and stable nursing workforce and supporting sustainable healthcare services [3].

The nursing work environment is an important modifiable organizational factor associated with nurses’ SWB [3, 6]. Favorable work environments may provide resources such as managerial support, interprofessional cooperation, recognition, autonomy, material assistance, career opportunities, and adequate staffing. However, the availability of a supportive work environment does not necessarily mean that all individuals derive the same psychological benefit from it. People working under similar organizational conditions may differ in how they perceive job demands and resources and in how these perceptions are related to work engagement and mental health [7]. From a Cognitive‐Affective Personality System (CAPS) perspective, such differences may arise because individuals differ in how they encode workplace cues and activate beliefs, affective responses, goals, outcome expectations, and self‐regulatory processes [8, 9]. These findings support the hypothesis that the nursing work environment is positively associated with SWB while also suggesting that the strength of this association may vary across individuals.

Previous studies have generally examined the nursing work environment and personality as separate predictors of psychological outcomes. Research on nursing work environments has shown that favorable organizational conditions are associated with better well‐being, lower burnout, and more favorable work‐related outcomes, but these studies commonly estimate an average association for the whole sample [3, 6, 10]. Personality research has primarily adopted a variable‐centered approach, examining the separate associations of individual Big Five traits—particularly neuroticism and extraversion—with psychological outcomes [11–14]. Such findings support a direct relationship between personality characteristics and well‐being, but they do not fully represent how multiple traits coexist and function together within the same person.

Person‐centered methods, including cluster analysis and latent profile analysis (LPA), address this limitation by identifying subgroups characterized by distinct configurations of personality traits [15–18]. Classic person‐centered studies have repeatedly described resilient, overcontrolled, and undercontrolled prototypes, although their exact replication and interpretation remain debated [19–23]. The resilient prototype is generally characterized by low neuroticism and relatively high levels of the other Big Five traits; the overcontrolled prototype is primarily characterized by higher neuroticism and lower extraversion; and the undercontrolled prototype is commonly characterized by lower agreeableness and conscientiousness [19–23]. These prototypes provide a theoretical framework for interpreting personality configurations identified among nurses, although profiles derived from a single nursing sample should not be regarded as personality types unique to the profession. Overall, existing research indicates that the nursing work environment is associated with SWB and that personality characteristics are also associated with SWB. However, it remains unclear whether person‐centered personality profiles alter the strength of the relationship between the nursing work environment and SWB.

CAPS theory provides the theoretical basis for this moderation hypothesis. According to CAPS, personality operates through an organized system of cognitive‐affective units, including encoding strategies, beliefs and expectations, goals, emotional responses, competencies, and self‐regulatory processes [8, 9]. When individuals encounter the same workplace condition, differences in the accessibility and activation of these units may lead them to assign different psychological meanings to that condition. A favorable work‐environment resource may therefore be interpreted as supportive, manageable, or rewarding by some nurses, but as evaluative, uncertain, or demanding by others. Based on the more adaptive trait configuration of the resilient prototype, favorable work‐environment dimensions were expected to show the strongest positive associations with SWB in a resilient‐like profile and weaker or less consistent associations in overcontrolled‐like and undercontrolled‐like profiles [19–23].

Guided by the CAPS theory, the present study addressed the insufficiently tested moderating role of person‐centered personality configurations in the relationship between the nursing work environment and SWB. Specifically, the study examined whether clinical nurses exhibited distinct Big Five personality profiles, whether nursing work‐environment dimensions were positively associated with SWB, and whether the strength of these associations differed across personality profiles. Based on previous evidence and the relatively adaptive trait configuration of the resilient prototype, these positive associations were expected to be strongest in a resilient‐like profile and weaker or less consistent in overcontrolled‐like and undercontrolled‐like profiles. Accordingly, three sets of hypotheses were tested. First, clinical nurses were expected to exhibit multiple distinct Big Five personality profiles, although the exact number and labels of the profiles were determined through LPA rather than imposed in advance. Second, the nursing work environment was hypothesized to be positively associated with SWB. Third, personality profiles were hypothesized to moderate these associations. After the profiles were identified, their correspondence with the classic resilient, overcontrolled, and undercontrolled prototypes was evaluated. A list of abbreviations used throughout this manuscript is provided in Table A1.

## 2. Background

SWB encompasses affective experiences, satisfaction with important life domains, and overall evaluations of life [24, 25]. It is particularly relevant in nursing because nurses frequently encounter emotional demands, heavy workloads, clinical risks, and repeated interpersonal stress, which are associated with emotional exhaustion, burnout, and poorer well‐being [1, 4, 5, 26]. Understanding the factors associated with nurses’ SWB is therefore important for protecting mental health and supporting workforce stability [3].

The nursing work environment represents the organizational and professional conditions that support or constrain nursing practice and is a multidimensional, potentially modifiable context [10, 27]. The Nursing Work Environment Scale used in this study was developed for Chinese hospitals and comprises seven dimensions: career development, leadership and management, nurse–physician relationships, recognition climate, professional autonomy, basic support, and staffing adequacy [28]. These dimensions were examined separately because they represent distinct management domains. Career development reflects opportunities for professional learning and advancement; leadership and management encompass managerial support, fairness, communication, and coordination; nurse–physician relationships reflect interprofessional cooperation and respect; recognition climate concerns acknowledgment of nurses’ contributions and professional value; professional autonomy reflects discretion and control over nursing practice; basic support concerns access to equipment, information, and organizational assistance; and staffing adequacy reflects whether sufficient personnel are available to meet clinical demands safely [28]. More favorable nursing work environments have generally been associated with better nurse well‐being and work‐related outcomes [3, 6, 10]. Nevertheless, nurses exposed to similar conditions may not experience identical psychological benefits, indicating the value of considering the personality system through which workplace resources are perceived and used [3, 7].

Within the Big Five framework, personality is described in terms of extraversion, agreeableness, conscientiousness, neuroticism, and openness [12, 13]. These traits can be analyzed separately, but they coexist within individuals and may form distinct configurations. Person‐centered methods have been used to identify such configurations and to examine whether they resemble established personality prototypes [15–19]. The resilient, overcontrolled, and undercontrolled prototypes provide a widely used, although debated, theoretical reference for understanding how combinations of traits relate to emotional adjustment and responses to external demands [19–23].

The resilient prototype is generally characterized by low neuroticism and relatively high extraversion, agreeableness, conscientiousness, and openness [19–23]. This configuration has been associated with more favorable emotional adjustment and well‐being [14, 21–23]. At the trait level, lower neuroticism is consistently associated with less psychological distress, whereas higher extraversion, conscientiousness, agreeableness, and openness are generally associated with more favorable well‐being outcomes [11, 13, 14]. The overcontrolled prototype is commonly characterized by higher neuroticism and lower extraversion and has been linked to negative affect, social inhibition, and sensitivity to uncertainty or evaluation [19–23]. The undercontrolled prototype is often characterized by lower agreeableness and conscientiousness and has been associated with weaker behavioral regulation and more externalizing or interpersonal difficulties [19–23]. From a CAPS perspective, these patterns may also be associated with differences in how people interpret and respond to environmental resources [7–9]. In this study, the profiles identified through LPA were interpreted with reference to these prototypes, while their number, characteristics, and labels were determined from the data rather than imposed in advance.

The CAPS provides a process‐oriented framework for explaining why individuals may respond differently to similar environmental conditions [8, 9]. CAPS conceptualizes personality as an organized system of interconnected cognitive‐affective units, including encoding strategies, expectancies and beliefs, goals and values, affective responses, and competencies and self‐regulatory strategies [8]. When individuals encounter a situational cue, the activation of these units influences how the situation is interpreted, which outcomes are anticipated, what emotions are experienced, and which responses are selected. From this perspective, personality coherence does not require identical behavior across situations; rather, it may be expressed through relatively stable, person‐specific “if‐then” situation‐response patterns [8, 29]. Subsequent research has shown that the same situation does not necessarily affect all individuals in the same way; its effects may differ in both magnitude and direction across individuals [30]. For example, individuals with stronger self‐regulatory capacity may experience fewer interpersonal difficulties and less impairment in psychological well‐being, even when they are highly sensitive to rejection [31]. Individuals who are more sensitive to identity‐related rejection may experience greater discomfort and distrust in institutional settings and may show poorer longer‐term adaptation [32]. In addition, ambiguous interpersonal events may produce stronger negative emotions when they are interpreted as being related to racial identity [33]. Collectively, these studies suggest that the psychological effects of environmental cues depend not only on the situation itself but also on how individuals interpret, anticipate, and regulate those cues. Consistent with this perspective, occupational research has shown that personality types are associated with perceptions of job demands and resources and with the relationships of these perceptions to work engagement and mental health [7].

In the present study, the seven nursing work‐environment dimensions were conceptualized as different types of workplace cues based on their scale definitions [28]. Career development may signal future opportunity; leadership and management may signal support, fairness, and trust; nurse–physician relationships may signal cooperation and interpersonal safety; recognition climate may signal professional value and evaluation; professional autonomy may signal control and responsibility; basic support may signal the availability of assistance; and staffing adequacy may signal workload and work control. Previous nursing research has generally treated favorable work environments as beneficial organizational resources and linked them with better nurse outcomes [3, 6, 10, 27]. Accordingly, each dimension was expected to be positively associated with SWB. CAPS proposes that individuals differ in how they encode situational cues and activate beliefs, expectations, affective responses, goals, and self‐regulatory processes [8, 9]. Applying this framework to nursing, the same work‐environment resource may carry different psychological meanings for nurses with different personality configurations. A resilient‐like configuration may be more likely to interpret favorable workplace cues as supportive and manageable, whereas an overcontrolled‐like configuration may respond more cautiously to uncertainty and evaluation; an undercontrolled‐like configuration may show less consistent responses because of lower agreeableness, conscientiousness, and behavioral self‐regulation [19–23]. These were theoretically derived expectations rather than processes directly measured in the present study. On this basis, the positive associations between work‐environment dimensions and SWB were hypothesized to be strongest in a resilient‐like profile and weaker or less consistent in overcontrolled‐like and undercontrolled‐like profiles.

### 2.1. Hypotheses

#### 2.1.1. Personality‐Profile Hypothesis

 H1: Clinical nurses will exhibit multiple distinct personality profiles based on their configurations of the Big Five traits. The exact number and labels of the profiles will be determined through LPA, although broad correspondence with resilient, overcontrolled, and undercontrolled prototypes is expected.

#### 2.1.2. Main‐Effect Hypotheses

 H2: More favorable nursing work‐environment conditions will be positively associated with nurses’ SWB.
 H2a: Career development will be positively associated with SWB.
 H2b: Leadership and management will be positively associated with SWB.
 H2c: Nurse–physician relationships will be positively associated with SWB.
 H2d: Recognition climate will be positively associated with SWB.
 H2e: Professional autonomy will be positively associated with SWB.
 H2f: Basic support will be positively associated with SWB.
 H2g: Staffing adequacy will be positively associated with SWB.

#### 2.1.3. Moderation Hypotheses

Across the following hypotheses, the positive association is expected to be strongest in a resilient‐like profile characterized by lower neuroticism and relatively higher levels of the other Big Five traits. It is expected to be weaker in an overcontrolled‐like profile characterized primarily by higher neuroticism and lower extraversion and weaker or less consistent in an undercontrolled‐like profile characterized primarily by lower agreeableness and conscientiousness. No exact ordering between the latter two profiles is proposed because their weaker associations are expected to arise through different psychological mechanisms.

 H3a: Personality profiles will moderate the positive association between career development and SWB. This association will be strongest in a resilient‐like profile and weaker in overcontrolled‐like and undercontrolled‐like profiles.
 H3b: Personality profiles will moderate the positive association between leadership and management and SWB. This association will be strongest in a resilient‐like profile and weaker in overcontrolled‐like and undercontrolled‐like profiles.
 H3c: Personality profiles will moderate the positive association between nurse–physician relationships and SWB. This association will be strongest in a resilient‐like profile and weaker in overcontrolled‐like and undercontrolled‐like profiles.
 H3d: Personality profiles will moderate the positive association between recognition climate and SWB. This association will be strongest in a resilient‐like profile and weaker in overcontrolled‐like and undercontrolled‐like profiles.
 H3e: Personality profiles will moderate the positive association between professional autonomy and SWB. This association will be strongest in a resilient‐like profile and weaker in overcontrolled‐like and undercontrolled‐like profiles.
 H3f: Personality profiles will moderate the positive association between basic support and SWB. This association will be strongest in a resilient‐like profile and weaker in overcontrolled‐like and undercontrolled‐like profiles.
 H3g: Personality profiles will moderate the positive association between staffing adequacy and SWB. This association will be strongest in a resilient‐like profile and weaker in overcontrolled‐like and undercontrolled‐like profiles.

Based on these hypotheses, Figure 1 presents the conceptual framework. The seven nursing work‐environment dimensions are treated as independent variables, Big Five personality profiles as the moderator, and SWB as the outcome.

**FIGURE 1:**
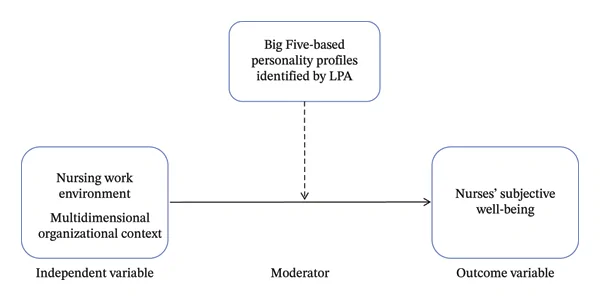
Conceptual framework of study.

## 3. Methods

This study employed a cross‐sectional, self‐report survey design and was reported in accordance with the Strengthening the Reporting of Observational Studies in Epidemiology (STROBE) statement [34]. As personality, perceptions of the nursing work environment, and SWB were measured concurrently, the analyses focused on associations rather than causal relationships. Reciprocal associations therefore cannot be ruled out; for example, nurses with lower SWB may be more likely to perceive their work environment negatively.

### 3.1. Study Design

This study employed a cross‐sectional survey design.

### 3.2. Study Participants

#### 3.2.1. Sampling Method

From May to August 2024, registered nurses from a tertiary Grade A hospital in Beijing were selected using convenience sampling. This hospital was selected because it is a large comprehensive tertiary hospital with a relatively large nursing workforce, complete clinical departments, and a relatively standardized nursing management system. These characteristics allowed the study to recruit nurses from different clinical units within a shared hospital‐level management context. We recognized that nurses’ work experiences may still vary across departments; therefore, nurses from different clinical departments were invited to participate, and department, together with other relevant demographic and work‐related variables, was included as a control variable in the subsequent hierarchical regression analyses. However, because this study used a single‐center convenience sampling design, the sample may have been influenced by hospital level, geographic region, organizational management characteristics, and nurses’ willingness to participate. Thus, potential sampling bias could not be fully eliminated, and the generalizability of the findings should be interpreted with caution.

#### 3.2.2. Inclusion and Exclusion Criteria

Inclusion criteria: ① currently employed as a registered nurse; ② engaged in clinical nursing work; ③ volunteer participation and informed consent provided.

Exclusion Criteria: ① nurses in training (interns, residents, or visiting nurses) who were not working independently; ② nurses absent during the survey period (e.g., sick leave, maternity leave, and business trips); ③ nurses with less than 1 year of work experience (because nurses in the early stage of practice are often still undergoing professional role transition, which may make their work roles and perceptions of the clinical work environment relatively unstable) [35].

#### 3.2.3. Sample Size

A total of 1509 questionnaires were collected. After screening, 323 questionnaires were excluded as invalid, and 1186 valid questionnaires were included in the final analysis. Questionnaires were considered invalid if they met any of the following criteria: ① the same response option was selected for almost all scale items, indicating uniform responses; ② obvious patterned responses were identified, such as repeated or regular response sequences; or ③ logical contradictions were found among related items. The screening was conducted independently by two researchers, and disagreements were resolved through discussion.

### 3.3. Research Instruments

#### 3.3.1. Demographic Questionnaire

The demographic questionnaire collected information on age, gender, department, education level, employment type, years of nursing experience, average number of night shifts per month, monthly income, and marital status.

#### 3.3.2. General Well‐Being (GWB) Schedule

The 18‐item GWB Schedule was originally developed for the first U.S. Health and Nutrition Examination Survey to assess individuals’ self‐reported psychological well‐being and distress. Fazio [36] subsequently evaluated its concurrent validity. Basic information on the 18‐item instrument, including its dimensional structure, response formats, reverse‐scored items, and scoring direction, is publicly available through the NIH Common Data Elements Repository maintained by the U.S. National Library of Medicine [37]. The instrument has been adopted by Chinese researchers and widely used in Chinese populations, demonstrating strong reliability, with Cronbach’s *α* coefficients of 0.95 for women and 0.91 for men and a test–retest reliability coefficient of 0.80 [38]. In the present study, SWB was operationally defined as participants’ overall subjective evaluation of their life circumstances and emotional experiences. Because the study focused on overall SWB rather than differences across specific dimensions, the total score of the 18‐item GWB was used to assess SWB, with higher scores indicating greater SWB.

In the present study, Cronbach’s *α* coefficient for the GWB Schedule was 0.830, indicating acceptable internal consistency. Confirmatory factor analysis of the six‐factor model yielded the following fit indices: *χ* ^2^ = 1339.07, df = 120, *χ* ^2^/df = 11.159, CFI = 0.896, TLI = 0.868, RMSEA = 0.093, and SRMR = 0.053. Overall, although some fit indices reached acceptable levels, the model fit remained at the borderline of acceptability. These findings suggest that the six‐dimensional structure of the GWB received only partial support in the present sample and that there remains room for improvement in model fit. Therefore, the present study primarily interpreted nurses’ overall SWB on the basis of the total GWB Schedule score, while the dimensional structure of the scale was interpreted with caution.

#### 3.3.3. Chinese Big Five Personality Inventory‐15 (CBF‐PI‐15)

To reduce participant burden, this study used the CBF‐PI‐15 revised by Zhang et al. [39]. The scale consists of 15 items and assesses five personality dimensions: extraversion, openness, agreeableness, conscientiousness, and neuroticism, with three items measuring each dimension. Higher scores indicate stronger expression of the corresponding personality trait. In the original validation study, Cronbach’s *α* coefficients for the five dimensions ranged from 0.61 to 0.80, indicating acceptable reliability for this brief personality measure.

In the present study, Cronbach’s *α* coefficients were 0.855 for extraversion, 0.917 for openness, 0.895 for agreeableness, 0.745 for conscientiousness, and 0.902 for neuroticism. The overall Cronbach’s *α* coefficient for the 15 items was 0.704. Confirmatory factor analysis showed that the correlated five‐factor model had an acceptable fit: *χ* ^2^ = 576.280, df = 80, *χ* ^2^/df = 7.204, CFI = 0.958, TLI = 0.945, RMSEA = 0.072, and SRMR = 0.075. Although the *χ* ^2^/df ratio was relatively high, the CFI, TLI, RMSEA, and SRMR values met commonly accepted criteria, indicating an acceptable overall model fit.

The average variance extracted (AVE) values for extraversion, openness, agreeableness, conscientiousness, and neuroticism were 0.692, 0.794, 0.741, 0.559, and 0.758, respectively. All AVE values exceeded 0.50, supporting adequate convergent validity. Discriminant validity was generally supported because the square roots of the AVE values exceeded most interfactor correlations. The latent correlation between conscientiousness and agreeableness was 0.766, which was slightly higher than the square root of the AVE for conscientiousness (0.748), suggesting some conceptual overlap. However, the HTMT value was 0.762, with a bootstrap 95% confidence interval of 0.707–0.814, which was below the recommended threshold of 0.85. Thus, the five dimensions demonstrated acceptable discriminant validity overall, although the distinction between conscientiousness and agreeableness was relatively weak. The scale was obtained from the supporting information published with the original open‐access article.

#### 3.3.4. Nursing Work Environment Scale

Several instruments, including the Nursing Work Index (NWI) and its revisions, have been used to assess nursing practice environments. Reviews have noted limitations related to cultural applicability, dated content, and instrument length [40]. Therefore, this study adopted the Nursing Work Environment Scale developed by Shao et al. [28] in 2016. The scale was specifically developed for the Chinese healthcare context and consists of 26 items across seven dimensions: career development, leadership and management, nurse–physician relationships, recognition climate, professional autonomy, basic support, and staffing adequacy. Higher scores indicate a more favorable nursing work environment. The original scale demonstrated excellent internal consistency, with Cronbach’s *α* coefficient of 0.946.

In the present study, the overall Cronbach’s *α* coefficient was 0.974. The *α* coefficients for career development, leadership and management, nurse–physician relationships, recognition climate, professional autonomy, basic support, and staffing adequacy were 0.934, 0.952, 0.944, 0.923, 0.916, 0.899, and 0.907, respectively. Confirmatory factor analysis showed that the correlated seven‐factor model had a generally acceptable fit: *χ* ^2^ = 2602.824, df = 278, *χ* ^2^/df = 9.370, CFI = 0.934, TLI = 0.923, RMSEA = 0.084, and SRMR = 0.046. Although the *χ* ^2^/df ratio was relatively high, and the RMSEA was slightly above 0.08, the CFI, TLI, and SRMR values met acceptable criteria, indicating a basically acceptable overall model fit. The AVE values for career development, leadership and management, nurse–physician relationships, recognition climate, professional autonomy, basic support, and staffing adequacy were 0.743, 0.841, 0.819, 0.800, 0.742, 0.759, and 0.775, respectively. All AVE values exceeded 0.50, supporting good convergent validity. Discriminant validity was supported for most dimensions because their square roots of AVE exceeded the corresponding interfactor correlations and most HTMT values were below 0.90. However, the latent correlation between basic support and staffing adequacy was 0.911, which exceeded the square roots of their AVE values, 0.871 and 0.880, respectively. Their HTMT value was 0.924, with a bootstrap 95% confidence interval of 0.898–0.950. Therefore, the seven‐factor structure was generally supported, although the discriminant validity between basic support and staffing adequacy should be interpreted with caution.

### 3.4. Statistical Analysis

Data were independently entered by two researchers and then exported for analysis using SPSS 26.0 and Mplus 8.3. Considering that the nursing work environment reflects external organizational conditions, whereas SWB reflects nurses’ overall psychological health status, the two variables were analyzed at different levels. Theoretically, the dimensions of the nursing work environment represent distinct and practically meaningful management areas; therefore, dimension‐level analysis can help identify specific environmental factors associated with nurses’ well‐being. In contrast, the content domains of the GWB Schedule jointly contribute to the assessment of GWB, and SWB was therefore treated as an overall outcome variable. Methodologically, analyzing SWB as multiple dimensional outcomes would require separate hypotheses and models for each domain and would increase the risk of multiple testing and complexity of interpretation. Therefore, nursing work environment dimension scores were entered as predictors, whereas the total score of SWB was used as the outcome variable.

Because all variables were assessed using self‐report questionnaires collected at the same time, Harman’s single‐factor test was conducted as a diagnostic assessment of potential common method variance. All items from the three instruments were entered into an unrotated exploratory factor analysis, and the proportion of variance explained by the first factor was examined. Because Harman’s test cannot conclusively rule out common method bias, its results were interpreted cautiously.

For LPA, the five Big Five personality dimension scores were standardized into z scores before model estimation to place all indicators on a comparable metric. The number of profiles was determined by considering model fit indices, classification quality, profile size, parsimony, and theoretical interpretability. Profile size was also used as an important criterion for model selection. Spurk et al. [16] suggested that an additional profile containing less than 1.0% of the total sample or fewer than 25 cases should generally be rejected, whereas Nylund‐Gibson et al. [41] noted that profiles comprising less than 5% of the sample should be interpreted cautiously and retained only when supported by sufficient sample size and substantive justification. Therefore, to ensure model stability and interpretability, profiles accounting for less than 5% of the total sample were not retained in this study.

In this study, the standardized mean scores of each personality dimension were used to describe the characteristics of each latent profile. To facilitate interpretation, a deviation of approximately 0.5 standard deviations from the sample mean was used as a descriptive reference, drawing on Cohen’s conventional interpretation of a standardized mean difference of 0.5 as a medium effect [42]. Accordingly, profile mean z scores above +0.5 were described as relatively high, whereas scores below −0.5 were described as relatively low. Scores between −0.5 and + 0.5 were not considered distinctly high or low and were interpreted according to their relative positions across profiles, the overall configuration of personality traits, and their theoretical meaning. Importantly, the ±0.5 criterion was used only as a descriptive heuristic to assist profile interpretation and labeling; it was not used to determine the number of profiles or to classify individuals. Individual profile membership was based on posterior class‐membership probabilities estimated by the LPA model, typically assigning each individual to the profile with the highest posterior probability, rather than on manual classification using z‐score cutoffs.

Following the moderation effect analysis method proposed by Wen Zhonglin [43], hierarchical regression analysis was performed to examine whether personality profiles moderated the associations between nursing work environment dimensions and SWB. Before creating interaction terms, continuous nursing work environment variables were mean‐centered. Personality profiles were dummy‐coded, with the resilient profile used as the reference group. Hardy [44] noted that dummy coding is appropriate when researchers have a clear control or comparison group in mind. Under this coding system, the regression coefficients for the other categories are interpreted as differences relative to the reference group. Hardy also suggested that a suitable reference group should meet three criteria: it should be a clearly established group rather than an “other” category; there should be a logical reason for selecting it as the comparison group, such as when the group is expected to score highest on the dependent variable; and its sample size should be substantive and not small compared with the other groups. Based on these methodological principles, the resilient profile was selected as the reference group in this study. The resilient profile was a clearly identified profile derived from the LPA model rather than a residual “other” category; it had a logical basis for comparison because it was characterized by lower neuroticism and relatively higher levels of the other Big Five traits, representing a theoretically meaningful adaptive comparison profile; and its sample size was sufficient to serve as a stable comparison baseline. Therefore, the coefficients for the cautious and independent profiles, as well as their interaction terms, were interpreted as differences relative to the resilient profile.

In the hierarchical regression models, control variables identified in the univariate analyses were entered in the first step. The mean‐centered nursing work‐environment dimension and personality‐profile dummy variables were entered in the second and third steps, respectively, followed by the complete block of interaction terms in the final step. The overall moderation effect in each model was evaluated using the change in *R* ^2^ and the corresponding F‐change test associated with the addition of the interaction‐term block. For interaction blocks that reached the conventional significance level, simple slope analyses were conducted by re‐estimating the final regression model with each personality profile successively specified as the reference category. In each reparameterized model, the unstandardized coefficient of the mean‐centered nursing work‐environment dimension represented the simple slope for the reference profile, and its standard error, t value, and *p* value were obtained directly from the regression output. The same control variables, predictors, and interaction terms were retained across models; only the reference category and the corresponding dummy‐variable coding were changed. Simple‐slope findings were interpreted in the context of the Bonferroni‐corrected omnibus interaction‐block test.

Because seven separate moderation models were tested, one for each dimension of the nursing work environment, the family‐wise type I error rate was controlled using the Bonferroni procedure. The conventional two‐sided significance level of 0.05 was divided by seven, resulting in a corrected significance threshold of *α* = 0.0071. The correction was applied to the seven omnibus F‐change tests for the interaction‐term blocks. Equivalently, Bonferroni‐adjusted *p* values were calculated as *P*_adjusted_ = min(7*P*_unadjusted_,  1). An overall moderation effect was considered statistically significant only when the unadjusted *p* value for the interaction block was below 0.0071, or equivalently, when the Bonferroni‐adjusted *p* value was below 0.05.

## 4. Results

### 4.1. Common Method Bias Test

Harman’s single‐factor test was conducted as a diagnostic assessment of potential common method variance. Ten factors had eigenvalues greater than 1, and the first factor accounted for 37.64% of the total variance, which was below the commonly used 40% criterion. These findings suggested that no single factor dominated the covariance among the measured variables. However, because all variables were collected through self‐report questionnaires at the same time, the possibility of common method bias could not be completely excluded.

### 4.2. General Demographic Characteristics

Among the 1186 nurses surveyed, 1071 (90.3%) were female, and 115 (9.7%) were male. Nurses aged between 30 and 40 years accounted for 538 cases (45.4%). A total of 927 nurses (78.2%) held a bachelor’s degree. Married nurses comprised 748 cases (63.1%). In terms of departments, surgical nurses had the highest proportion, with 305 cases (25.7%). The largest group by professional title was nurses with the title of Nurse Practitioner, totaling 710 cases (59.9%). Most nurses had 11–20 years of work experience, accounting for 424 cases (35.8%). Regarding monthly income, the largest group earned between 8001 and 12,000 CNY, comprising 545 cases (46.0%). The majority of nurses were formally employed within the staffing system, with 696 cases (58.7%). Details are shown in Table 1.

**TABLE 1:** General demographic characteristics (*n* = 1186).

Item	Category	*n*	%
Gender	Male	115	9.7
	Female	1071	90.3

Age	< 25 years	114	9.6
	25–29 years	311	26.2
	30–40 years	538	45.4
	> 40 years	223	18.8

Education level	Secondary nursing school	9	0.8
	Junior college	239	20.2
	Bachelor’s degree	927	78.2
	Master’s degree or above	11	0.9

Marital status	Single	414	34.9
	Married	748	63.1
	Divorced	22	1.9
	Widowed	2	0.2

Department	Internal medicine	262	22.1
	Surgery	305	25.7
	Obstetrics and gynecology	72	6.1
	Pediatrics	54	4.6
	Intensive care unit	87	7.3
	Emergency department	50	4.2
	Outpatient	160	13.5
	Operating room	196	16.5

Professional title	Nurse	246	20.7
	Nurse practitioner	710	59.9
	Senior nurse practitioner	223	18.8
	Associate chief nurse or above	7	0.6

Years of service	1–4 years	234	19.7
	5–10 years	363	30.6
	11–20 years	424	35.8
	> 20 years	165	13.9

Monthly income	≤ 4000 CNY	8	0.7
	4001–8000 CNY	100	8.4
	8001–12,000 CNY	545	46.0
	> 12,000 CNY	533	44.9

Employment type	Officially employed	696	58.7
	Contract‐based	490	41.3

### 4.3. Analysis of Differences in Nurses’ SWB Across General Demographic Characteristics

The results indicate that SWB scores differed significantly by age (*F* = 3.72, *p* < 0.05), department (*F* = 7.979, *p* < 0.001), years of work experience (*F* = 2.96, *p* < 0.05), marital status (*F* = 3.99, *p* < 0.01), and employment type (*t* = 3.391, *p* < 0.001). These findings suggest that nurses’ SWB was not only related to individual psychological characteristics, but was also embedded in their professional stage, clinical work context, family status, and employment status. Specifically, age and years of work experience may reflect differences in career stage and accumulated work adaptation; department may capture variation in clinical workload, patient characteristics, and unit‐level work demands; marital status may reflect differences in family and social support; and employment type may be related to perceived job security and organizational belonging.

Therefore, these variables were considered important contextual factors that could influence nurses’ SWB. To reduce their potential confounding effects, age, department, years of work experience, marital status, and employment type were included as control variables in the subsequent hierarchical regression analyses. This approach helped clarify whether personality profiles and nursing work environment dimensions were associated with SWB beyond the influence of these demographic and work‐related factors. Details are shown in Supporting Table S1.

### 4.4. Classification of Nurses’ Personality Types

This study conducted an LPA on the Big Five personality trait scores of 1186 nurses, revealing the underlying personality type combination patterns in the nursing population from a holistic perspective and laying the groundwork for further exploring the moderating role of personality types.

#### 4.4.1. Results of the LPA

The Big Five personality scores were converted to Z‐scores before conducting the LPA. To determine the optimal number of classes, models with 1–6 potential classes were sequentially fitted and evaluated. The three‐class model showed the best performance in terms of Entropy, indicating the highest classification precision. Although AIC, BIC, and aBIC values decreased with more classes, the LMR test became nonsignificant from the three‐to five‐class models, suggesting that additional classes did not substantially improve fit. Considering all indices, the three‐class model offered the best balance between model fit and classification clarity and was selected as the final model (Supporting Table S2). Average posterior class‐membership probabilities indicated good classification quality. The average probabilities of membership in the assigned profiles were 88.5% for Class 1, 90.5% for Class 2, and 95.4% for Class 3. The corresponding off‐diagonal probabilities were 11.4% and 0.1% for Class 1 (membership in Classes 2 and 3, respectively), 8.6% and 1.0% for Class 2 (membership in Classes 1 and 3, respectively), and 0% and 4.6% for Class 3 (membership in Classes 1 and 2, respectively), supporting adequate separation among the three profiles (Supporting Table S3).

#### 4.4.2. Naming of Personality Types

As shown in Figure 2, the specific performance of each personality type in each trait dimension is observable.

**FIGURE 2:**
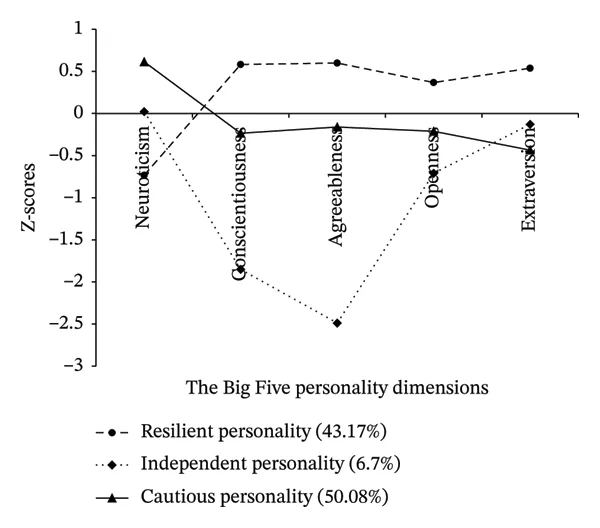
Characteristic profiles of latent classes of nurse personality types.

Class 1 included 512 nurses, accounting for 43.17% of the total sample. This group showed a standardized neuroticism score below −0.5, indicating a relatively low level. In contrast, standardized scores for conscientiousness, agreeableness, and extraversion were above 0.5, while openness was between 0 and 0.5. The profile was therefore characterized by emotional stability, stronger self‐regulation, positive interpersonal orientation, and higher social expressiveness. This pattern closely resembled the classic resilient prototype, which is generally characterized by low neuroticism and relatively high levels of the other Big Five traits [19–23]. Accordingly, this profile was named “Resilient Personality.”

Class 2 included 594 nurses, accounting for 50.08% of the total sample. This group showed a standardized neuroticism score above 0.5, while the other trait scores ranged between 0 and ‐0.5; extraversion was the lowest among the three profiles. This pattern indicated relatively high emotional sensitivity and a restrained interpersonal style. Profiles with higher neuroticism and lower extraversion have often been interpreted as resembling the overcontrolled prototype [19–23]. However, because the term “overcontrolled” may carry a negative or pathologizing implication, the more neutral label “Cautious Personality” was used to reflect emotional vigilance and reserved social expression.

Class 3 included 80 nurses, accounting for 6.75% of the total sample. Neuroticism was close to the sample mean, extraversion was moderately low, and conscientiousness, agreeableness, and openness were relatively low; agreeableness and conscientiousness were the lowest among the three profiles. Lower agreeableness may reflect less interpersonal accommodation, whereas lower conscientiousness may indicate weaker rule‐oriented self‐regulation and task organization. This pattern partially resembled the undercontrolled prototype, which is commonly characterized by relatively low agreeableness and conscientiousness [19–23]. Because neuroticism was not markedly high and the main features suggested lower compliance and a more self‐directed interpersonal orientation, the profile was named “Independent Personality.”

### 4.5. The Moderating Role of Personality Types in the Impact of the Nursing Work Environment on Nurses’ SWB

Hierarchical regression was used to examine whether personality profiles moderated the associations between seven nursing work environment dimensions and nurses’ SWB. Moderation effects were observed only for recognition climate and professional autonomy. For career development, leadership and management, nurse–physician relationships, basic support, and staffing adequacy, the interaction terms were not statistically significant (Supporting Tables S4 and S5). These results suggest that the moderating role of personality profiles was specific to selected work environment dimensions rather than universal across all dimensions.

#### 4.5.1. Testing the Moderating Effect of Personality Types on the Relationship Between Recognition Climate and Nurses’ SWB

The interaction terms between recognition climate and the two personality‐profile dummy variables were entered in the final step of the hierarchical regression model. The final model explained 46.0% of the variance in SWB (*R* ^2^ = 0.460, adjusted *R* ^2^ = 0.450). Adding the interaction block explained an additional 0.6% of the variance, Δ*R* ^2^ = 0.006, ΔF = 6.355, *p* = 0.002. This *p* value was below the Bonferroni‐corrected significance threshold of *α* = 0.0071 for the seven omnibus moderation tests. Thus, personality profile significantly moderated the association between recognition climate and SWB after correction for multiple testing.

The moderation pattern was mainly attributable to the difference between the cautious and resilient profiles. The recognition climate × cautious profile interaction was negative and statistically significant (*B* = −1.538, SE = 0.438, *β* = −0.140, *t* = −3.509, *p* < 0.001), indicating that the positive association between recognition climate and SWB was significantly weaker among nurses with the cautious profile than among those with the resilient profile. By contrast, the recognition climate × independent profile interaction was not statistically significant (*B* = −0.782, SE = 0.532, *β* = −0.048, *t* = −1.471, *p* = 0.142), indicating that the strength of the association did not differ significantly between nurses with the independent and resilient profiles. The detailed results of the moderation analysis for recognition climate are presented in Table 2.

**TABLE 2:** Test of the moderating effect of personality type on the relationship between recognition climate and nurses’ subjective well‐being (*n* = 1186).

**Model**	**R** ^2^	**Adjusted** **R** ^2^	**Δ** **R** ^2^	**Δ*F***	

*Panel A. Hierarchical model fit*					
1. Covariates	0.077	0.064	0.077	5.770	
2. +Work‐environment dimension	0.275	0.264	0.198	318.353	
3. +Personality‐profile indicators	0.454	0.445	0.179	191.354	
4. +Personality‐profile interactions	0.460	0.450	0.006	6.355	

**Predictor**	***B***	**SE**	***β***	**95% CI for *B***	***p***

*Panel B. Final-model focal effects (Model 4)*					
Recognition climate	3.310	0.366	0.435	2.592∼4.028	< 0.001
Cautious personality vs. resilient	−11.842	0.662	−0.447	−13.141∼−10.542	< 0.001
Independent personality vs. resilient	−10.029	1.334	−0.190	−12.647∼−7.412	< 0.001
Recognition climate × cautious	−1.538	0.438	−0.140	−2.397∼−0.678	< 0.001
Recognition climate × independent	−0.782	0.532	−0.048	−1.825∼0.261	0.142

*Note:* Outcome: nurses’ subjective well‐being; *n* = 1186. *B* = unstandardized regression coefficient; *β* = standardized regression coefficient; Δ*F* = change‐in‐F statistic for each model step. The resilient personality profile was used as the reference category. Bonferroni correction was applied to the seven omnibus moderation tests, with a corrected significance threshold of *α* = 0.05/7 ≈ 0.0071. The seven‐model comparison and focal final‐model coefficients are provided in Supporting Tables S4 and S5, respectively. Simple slopes are reported in Supporting Table S6.

Abbreviations: CI = confidence interval, SE = standard error.

Simple slope analyses further characterized this pattern. Recognition climate was positively associated with SWB across all three personality profiles. Among nurses with the resilient profile, the simple slope was *B* = 3.310, SE = 0.366, *t* = 9.044, *p* < 0.001. Among nurses with the cautious profile, the simple slope was *B* = 1.773, SE = 0.246, *t* = 7.193, *p* < 0.001. Among nurses with the independent profile, the simple slope was *B* = 2.528, SE = 0.392, *t* = 6.457, *p* < 0.001. As shown in Figure 3, the resilient profile had the largest estimated positive slope, the independent profile showed an intermediate positive slope, and the cautious profile showed the smallest positive slope. Although recognition climate was positively associated with SWB within all three personality profiles, only the difference in slope between the cautious and resilient profiles was statistically significant; the slope for the independent profile did not differ significantly from that for the resilient profile. Detailed simple‐slope estimates are provided in Supporting Table S6.

**FIGURE 3:**
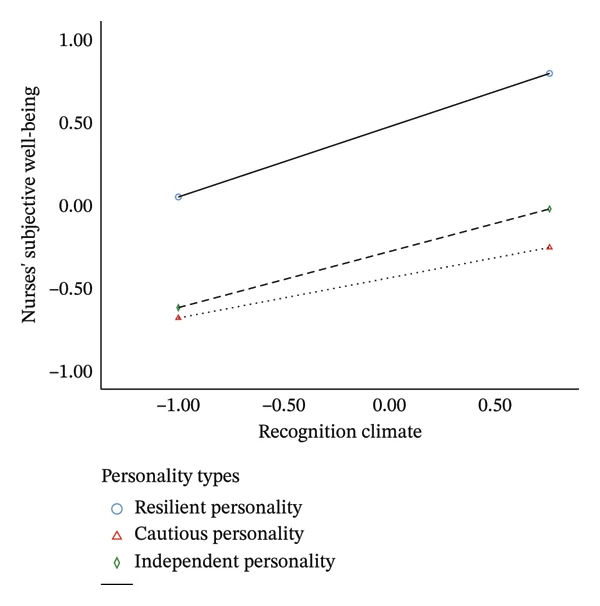
Simple slope plot of the relationship between recognition climate and nurses’ subjective well‐being across personality types.

Overall, these results provide partial support for H3d. Personality profile significantly moderated the association between recognition climate and SWB after correction for multiple testing. More specifically, the positive association between recognition climate and SWB was significantly weaker among nurses with the cautious personality profile than among those with the resilient personality profile. Recognition climate was also positively associated with SWB among nurses with the independent profile, but the strength of this association did not differ significantly from that observed among nurses with the resilient profile.

#### 4.5.2. Testing the Moderating Effect of Personality Types on the Relationship Between Professional Autonomy and Nurses’ SWB

The interaction terms between professional autonomy and the two personality‐profile dummy variables were entered in the final step of the hierarchical regression model. The final model explained 43.8% of the variance in SWB (*R* ^2^ = 0.438, adjusted *R* ^2^ = 0.427). Adding the interaction block explained an additional 0.5% of the variance, Δ*R* ^2^ = 0.005, ΔF = 5.207, *p* = 0.006. This *p* value was below the Bonferroni‐corrected significance threshold of *α* = 0.0071 for the seven omnibus moderation tests. Therefore, the overall moderating effect of personality profile on the relationship between professional autonomy and SWB remained statistically significant after correction for multiple testing.

At the coefficient level, the interaction between professional autonomy and the cautious personality profile was negative and statistically significant (*B* = −0.938, SE = 0.308, *β* = −0.121, *t* = −3.044, *p* < 0.01), indicating that the positive association between professional autonomy and SWB was significantly weaker among nurses with the cautious profile than among those with the resilient profile. In contrast, the interaction between professional autonomy and the independent personality profile was not statistically significant (*B* = −0.263, SE = 0.404, *β* = −0.021, *t* = −0.653, *p* = 0.514), indicating that the strength of the association between professional autonomy and SWB did not differ significantly between nurses with the independent and resilient profiles. The detailed results of the moderation analysis for professional autonomy are presented in Table 3.

**TABLE 3:** Test of the moderating effect of personality type on the relationship between professional autonomy and nurse’s subjective well‐being (*n* = 1186).

**Model**	**R** ^2^	**Adjusted** **R** ^2^	**Δ** **R** ^2^	**Δ*F***	

*Panel A. Hierarchical model fit*					
1. Covariates	0.077	0.064	0.077	5.770	
2. +Work‐environment dimension	0.234	0.222	0.156	238.237	
3. +Personality‐profile indicators	0.433	0.423	0.199	204.293	
4. +Personality‐profile interactions	0.438	0.427	0.005	5.207	

**Predictor**	***B***	**SE**	***β***	**95% CI for *B***	***p***

*Panel B. Final-model focal effects (Model 4)*					
Professional autonomy	1.944	0.254	0.353	1.446∼2.443	< 0.001
Cautious personality vs. resilient	−12.540	0.663	−0.474	−13.841∼−11.239	< 0.001
Independent personality vs. resilient	−11.149	1.352	−0.211	−13.801∼−8.497	< 0.001
Professional autonomy × cautious	−0.938	0.308	−0.121	−1.543∼−0.333	0.002
Professional autonomy × independent	−0.263	0.404	−0.021	−1.055∼0.528	0.514

*Note:* Outcome: nurses’ subjective well‐being; *n* = 1186. *B* = unstandardized regression coefficient; *β* = standardized regression coefficient; Δ*F* = change‐in‐*F* statistic for each model step. The resilient personality profile was used as the reference category. Bonferroni correction was applied to the seven omnibus moderation tests, with a corrected significance threshold of *α* = 0.05/7 ≈ 0.0071. The seven‐model comparison and focal final‐model coefficients are provided in Supporting Tables S4 and S5, respectively. Simple slopes are reported in Supporting Table S6.

Abbreviations: CI = confidence interval, SE = standard error.

Simple slope analyses showed that professional autonomy was positively associated with SWB across all three personality profiles. Among nurses with the resilient profile, the simple slope was *B* = 1.944, SE = 0.254, *t* = 7.652, *p* < 0.001. Among nurses with the cautious profile, the simple slope was *B* = 1.006, SE = 0.177, *t* = 5.677, *p* < 0.001. Among nurses with the independent profile, the simple slope was *B* = 1.681, SE = 0.316, *t* = 5.317, *p* < 0.001. As shown in Figure 4, the resilient profile had the largest estimated positive slope, followed by the independent profile, whereas the cautious profile had the smallest positive slope. Thus, although professional autonomy was positively associated with SWB within all three personality profiles, the strength of this association varied across personality profiles. Specifically, the positive association was significantly weaker among nurses with the cautious profile than among those with the resilient profile, whereas the slope for the independent profile did not differ significantly from that for the resilient profile. Detailed simple‐slope estimates are provided in Supporting Table S6.

**FIGURE 4:**
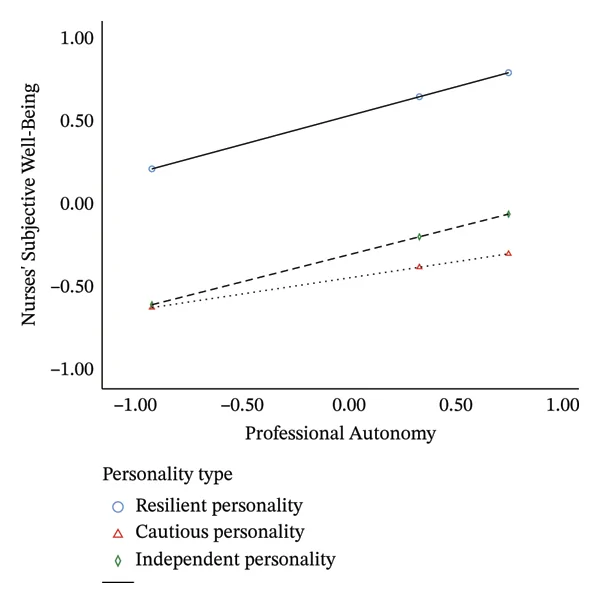
Simple slope plot of the relationship between professional autonomy and nurses’ subjective well‐being across personality types.

Overall, H3e was supported after correction for multiple testing. The omnibus interaction‐block test remained statistically significant after Bonferroni correction, indicating that personality profile moderated the relationship between professional autonomy and nurses’ SWB. More specifically, the positive association between professional autonomy and SWB was significantly weaker among nurses with the cautious personality profile than among those with the resilient personality profile, whereas no statistically significant difference was observed between the independent and resilient personality profiles.

### 4.6. Summary of Hypothesis Testing

Overall, H1 was supported, as the LPA identified three distinct personality profiles—resilient, cautious, and independent—with broad correspondence to the classic resilient, overcontrolled, and undercontrolled prototypes. H2a–H2g were supported because all seven nursing work‐environment dimensions were positively associated with SWB in the corresponding regression models. Regarding the moderation hypotheses, H3d was partially supported, and H3e was supported after Bonferroni correction. Personality profile significantly moderated the associations of both recognition climate and professional autonomy with SWB: in each model, the positive association was weaker among cautious nurses than among resilient nurses, whereas the slope for independent nurses did not differ significantly from that for resilient nurses. H3a, H3b, H3c, H3f, and H3g were not supported because no significant moderation was found for career development, leadership and management, nurse–physician relationships, basic support, or staffing adequacy.

## 5. Discussion

### 5.1. Personality Profiles and SWB Among Clinical Nurses

This study identified three Big Five personality profiles among clinical nurses: resilient, cautious, and independent. The resilient profile, comprising 43.17% of the sample, was characterized by relatively low neuroticism, high conscientiousness, agreeableness, and extraversion, and moderately high openness. This pattern showed the closest correspondence with the classic resilient prototype [19–23]. The cautious profile was the largest group, accounting for 50.08% of the sample, and was characterized by relatively high neuroticism and lower levels of the other traits, particularly extraversion. Although it partially resembled the overcontrolled prototype, the neutral label “cautious” was used because the profile primarily reflected emotional vigilance, sensitivity to uncertainty, and restrained interpersonal expression rather than dysfunction [19–23]. The independent profile accounted for 6.75% of the sample and was characterized by relatively low conscientiousness, agreeableness, and openness, moderately low extraversion, and neuroticism close to the sample mean. Its relatively low agreeableness and conscientiousness partially resembled the classic undercontrolled prototype. However, an important difference was that neuroticism in the independent profile remained close to the sample average rather than showing the relatively elevated level commonly associated with an undercontrolled pattern. Therefore, this profile should not be interpreted as indicating generalized behavioral dysregulation or a complete lack of self‐control. The label “independent” was adopted because it more appropriately reflected the profile’s relatively self‐directed, less accommodating, and less rule‐oriented disposition without pathologizing its members [19–23]. These findings support the value of a person‐centered approach by showing that multiple personality traits coexist in meaningful configurations rather than operating only as isolated characteristics. At the same time, the correspondence with the classic prototypes was approximate rather than exact. Because the participants were recruited from a single healthcare institution, the identified profiles should be interpreted as sample‐based configurations rather than personality types specific to the nursing profession.

The personality profiles also differed in their overall levels of SWB. Across the regression models, nurses with cautious and independent profiles had lower adjusted SWB than those with the resilient profile. This pattern is broadly consistent with evidence that lower neuroticism and higher extraversion, agreeableness, conscientiousness, and openness are associated with more favorable well‐being outcomes [11, 13, 14, 22, 23]. Lower neuroticism may be related to less negative affect and fewer threat‐oriented appraisals, while higher extraversion and agreeableness may facilitate positive emotion, interpersonal engagement, and access to social support. Higher conscientiousness and openness may support goal‐directed coping and flexible adaptation. Conversely, the higher neuroticism and lower extraversion of the cautious profile may be associated with greater emotional sensitivity and fewer positive emotional experiences, while the lower agreeableness and conscientiousness of the independent profile may be related to less consistent self‐regulation and use of interpersonal resources.

This finding extends trait‐based research by suggesting that personality configurations may provide additional information about nurses’ SWB. However, these explanations remain theoretical and should not be interpreted causally. The independent profile accounted for only 6.75% of the sample. Its low prevalence may partly reflect occupational attraction, selection, and retention processes, through which individuals are more likely to enter and remain in settings compatible with their characteristics [45]. Because clinical nursing relies heavily on coordination, communication, and teamwork [46], a profile characterized by relatively low agreeableness and conscientiousness may be less aligned with some collaborative demands [47]. Nevertheless, this interpretation is speculative. As the profile included only 80 nurses from a single hospital, its stability and occupational relevance require confirmation in larger, multicenter samples.

### 5.2. Selective Moderation Across Nursing Work‐Environment Dimensions

The moderation results showed a selective rather than universal pattern. The interaction blocks involving recognition climate and professional autonomy reached conventional levels of statistical significance, whereas no significant moderation was found for career development, leadership and management, nurse–physician relationships, basic support, or staffing adequacy. After Bonferroni correction for the seven moderation tests, both the recognition‐climate interaction (*p* = 0.002) and the professional‐autonomy interaction (*p* = 0.006) remained statistically significant. It suggests that personality profiles may be more relevant to nurses’ responses to certain workplace cues than to the nursing work environment as a whole. By examining the seven work‐environment dimensions separately and controlling the family‐wise type I error rate, the study provides a more differentiated and statistically cautious account of when personality‐related differences may emerge.

CAPS theory offers a process‐based explanation for this pattern [8, 9]. According to CAPS, individuals encode and interpret situational cues, which then activate beliefs, outcome expectancies, goals, affective responses, and self‐regulatory strategies. Because personality configurations may differ in the accessibility and activation of these cognitive‐affective units, the same workplace cue may produce different “if‐then” response patterns [8, 9, 29–33]. Recognition climate and professional autonomy convey information about professional value, control, trust, and responsibility and may therefore be particularly open to personality‐related interpretation. After correction for multiple testing, both recognition climate and professional autonomy received statistically significant support as personality‐sensitive workplace cues.

### 5.3. Recognition Climate as a Personality‐Sensitive Workplace Cue

Recognition climate was one of two work‐environment dimensions for which the overall interaction remained statistically significant after correction for multiple testing. Recognition climate communicates whether nurses’ efforts and professional contributions are noticed and valued and may activate self‐evaluation, perceived competence, expectations about others’ responses, and threat appraisal. The positive association between recognition climate and SWB was weaker among cautious nurses than among resilient nurses. Resilient nurses may be more likely to interpret recognition as affirmation and organizational support. By contrast, the higher neuroticism and lower extraversion of cautious nurses may make them more sensitive to evaluation, uncertainty, and possible negative consequences [11, 14, 19–23]. Recognition may therefore be interpreted not only as support but also as scrutiny, interpersonal comparison, or increased expectations. A strength of this finding is that it refines the common assumption that recognition functions as a uniformly positive organizational resource. The results suggest that the psychological value of recognition may depend partly on how it is delivered and interpreted. This does not mean that recognition is harmful to cautious nurses; rather, its benefits may be less consistent when it is experienced as evaluation or pressure.

Among nurses with the independent profile, recognition climate was also significantly and positively associated with SWB, but the slope did not differ significantly from that of the resilient profile. Thus, the study identified a positive within‐profile association without establishing a difference in its strength relative to the resilient profile. The relatively low agreeableness of the independent profile may reflect a more self‐directed interpersonal orientation [13, 22, 23]. Such nurses may place greater emphasis on recognition as direct feedback regarding task completion, individual contribution, and competence. Kerber et al. [23] also discussed lower delay of gratification as a possible feature of undercontrolled‐like profiles, which may imply greater responsiveness to immediate feedback and reward. Because the independent subgroup was small, this interpretation should remain tentative.

### 5.4. Professional Autonomy as a Personality‐Sensitive Workplace Cue

The moderating effect of personality profiles on the association between professional autonomy and SWB remained statistically significant after Bonferroni correction (Δ*R* ^2^ = 0.005, *p* = 0.006); therefore, H3e was supported. The positive association between professional autonomy and SWB was significantly weaker among cautious nurses than among resilient nurses, whereas no significant difference was detected between independent and resilient nurses. Professional autonomy remained positively associated with SWB within all three personality profiles, but the strength of the association differed mainly because of the cautious‐resilient contrast.

One possible explanation is that professional autonomy may carry different psychological meanings for different nurses. Autonomy may represent organizational trust, professional discretion, competence, and control, but it may also involve greater responsibility, uncertainty, and accountability. Occupational research suggests that personality types are associated with how individuals perceive job demands and resources [7]. Resilient nurses, characterized by lower neuroticism and relatively higher conscientiousness and other adaptive traits, may be more likely to view autonomy as a manageable work resource and an opportunity to exercise professional judgment [13, 19, 22, 23]. In contrast, the higher neuroticism and lower extraversion of cautious nurses may increase their attention to possible errors, external evaluation, and the consequences of independent decisions, thereby reducing the potential psychological benefits of autonomy [13, 22, 23].

### 5.5. General Work‐Environment Resources and the Appropriate Role of Personality

The absence of significant moderation for career development, leadership and management, nurse–physician relationships, basic support, and staffing adequacy may reflect their role as general job resources rather than personality‐diagnostic cues. CAPS and trait activation theory suggest that personality moderation is more likely when situational cues activate different expectations, emotions, goals, or self‐regulatory responses across individuals [8, 46]. These five dimensions may primarily provide broadly valued resources, such as support, coordination, security, and the capacity to meet work demands [47]. Because such resources address occupational needs shared across personality profiles, their associations with SWB may be relatively similar and therefore may not produce detectable moderation. Compared with recognition and autonomy, they may also contain fewer self‐evaluative or interpretively open cues. However, this explanation remains theoretical because the relevant cognitive–affective processes were not directly measured.

The magnitude of the significant interaction effects should also be interpreted cautiously. The interaction block involving recognition climate explained an additional 0.6% of the variance in SWB, whereas the interaction block involving professional autonomy explained an additional 0.5%. Given the sample size of 1186 nurses, statistical significance should not be equated with substantial practical importance, because effects of limited practical magnitude may still reach statistical significance. The small incremental contribution of the interaction terms helps define the appropriate role of personality profiles: they should not be regarded as major determinants of nurses’ responses to the work environment, but they may provide modest additional information about how particular workplace cues are interpreted and processed.

## 6. Limitations

Although this study included a relatively large sample of 1186 nurses and tested theory‐based hypotheses, its cross‐sectional design prevented causal inference. Future studies should use longitudinal or intervention designs to clarify the direction of the associations. Second, although Harman’s single‐factor test did not indicate a dominant factor, all variables were measured using self‐report questionnaires at the same time, and the affective content shared by neuroticism and the GWB may have inflated their association. Future research should combine self‐reports with objective indicators, supervisor ratings, or temporally separated measurements. Third, despite the substantial sample size, participants were recruited from a single tertiary hospital through convenience sampling, limiting generalizability. Multicenter studies involving hospitals of different levels and regions are therefore needed. Fourth, the independent profile included only 80 nurses (6.75%), which reduced the precision of subgroup estimates. Larger multicenter samples and simulation‐based power analyses should be used to ensure adequate numbers in smaller profiles. Finally, although examining seven work‐environment dimensions provided a detailed assessment, multiple testing increased the risk of false‐positive findings. After Bonferroni correction, both the recognition‐climate interaction (Δ*R* ^2^ = 0.006, *p* = 0.002) and the professional‐autonomy interaction (Δ*R* ^2^ = 0.005, *p* = 0.006) remained statistically significant. However, their incremental effects were small, and both findings require independent replication.

## 7. Implications

The findings suggest that the absence of significant moderation for career development, leadership and management, nurse–physician relationships, basic support, and staffing adequacy does not mean that these dimensions are unimportant. Rather, they may function as broadly beneficial organizational resources across personality profiles and should remain central priorities in nursing management.

The significant findings for recognition climate and professional autonomy suggest that nurses may differ in how they respond to evaluative cues and autonomy. Managers may therefore provide recognition that is specific, timely, supportive, and focused on professional contributions rather than interpersonal comparison. Similarly, autonomy may be more beneficial when accompanied by clear role expectations, defined decision boundaries, procedural guidance, and accessible supervisory support.

However, these implications should be interpreted cautiously because the moderation effects were small, explaining only 0.6% and 0.5% of additional variance in SWB. Personality‐informed support should therefore be regarded as a flexible supporting strategy rather than a substitute for adequate staffing, supportive leadership, teamwork, material support, and career‐development opportunities. Personality profiles should not be used for employee screening, restricting opportunities, or making fixed judgments about nurses; instead, they may help managers provide more flexible forms of feedback, guidance, and support.

## 8. Conclusion

This study identified resilient, cautious, and independent personality profiles among clinical nurses. The moderating role of personality profiles was selective rather than universal. After correction for seven moderation tests, the interactions involving both recognition climate and professional autonomy remained statistically significant. For both work‐environment dimensions, the positive association with SWB was weaker among cautious nurses than among resilient nurses, whereas independent nurses did not differ significantly from resilient nurses.

The recognition‐climate interaction explained an additional 0.6% of the variance in SWB, and the professional‐autonomy interaction explained an additional 0.5%, indicating small incremental effects. Findings involving the independent profile were also limited by the small size of this subgroup. The results therefore suggest that personality types may provide modest additional insight into how nurses interpret selected work‐environment cues, particularly recognition climate and professional autonomy. However, universal improvement of the nursing work environment should remain the principal management strategy, while personality‐informed support should be used cautiously as a supporting approach.

## Author Contributions

Baohua Li, Mingxuan Wang, Peitao Li, and Rongmei Geng conceived and designed the study; Mingxuan Wang and Xianjing Hu collected the data and analyzed the data; Baohua Li and Mingxuan Wang led the manuscript writing.

## Funding

This work was supported by the Nurses Health Cohort Research awarded by Peking University Third Hospital cohort construction project (BYSYDL2024004).

## Ethics Statement

This study was approved by the Institutional Review Board of Peking University Third Hospital (approval number: IRB00006761‐M2024552). The study was conducted in accordance with the principles of the Declaration of Helsinki. Written informed consent was obtained from all participants prior to data collection. All data were anonymized to protect participant confidentiality, and participants were informed of their right to withdraw from the study at any time without penalty.

## Conflicts of Interest

The authors declare no conflicts of interest.

## Supporting Information

Additional supporting information can be found online in the Supporting Information section.

## Supporting information


**Supporting Information** Supporting Table S1. Subjective well‐being by demographic characteristics. Supporting Table S2. Latent‐profile model fit indices. Supporting Table S3. Average posterior profile‐membership probabilities. Supporting Table S4. Hierarchical moderation model fit across seven work‐environment dimensions. Supporting Table S5. Final‐model focal coefficients across work‐environment dimensions. Supporting Table S6. Simple slopes of significant personality‐profile interactions.

## Data Availability

The data that support the findings of this study are available on request from the corresponding author. The data are not publicly available due to privacy or ethical restrictions.

## Appendix 1

**Table A1:** List of abbreviations.

Abbreviation	Full form
LPA	Latent profile analysis
SWB	Subjective well‐being
CAPS	Cognitive‐Affective Personality System
STROBE	Strengthening the Reporting of Observational Studies in Epidemiology
CBF‐PI‐15	Chinese Big Five Personality Inventory‐15
NWI	Nursing Work Index
