# Supplementary material for: How Personality Types Shape the Link Between Nursing Work Environment and Subjective Well‐Being
Source: J Nurs Manag. 2026 Nov 4;2026:1147669. doi: 10.1155/jonm/1147669 (PMC13736069; doi:10.1155/jonm/1147669)
Supplement: Supplementary file 1 [file JONM-2026-1147669-s001.docx]

Supplementary Table S1. Subjective well-being by demographic characteristics

| **Characteristic** | **Category** | **Well-being, mean ± SD** | **t/F** | **p** |
| --- | --- | --- | --- | --- |
| Gender | Male | 83.77±13.48 | -1.68 | 0.094 |
|  | Female | 85.94±13.20 | — | — |
| Age | <25 years | 86.68±13.82 | 3.72 | **0.011** |
|  | 25-29 years | 86.75±12.51 | — | — |
|  | 30-40 years | 86.00±13.36 | — | — |
|  | >40 years | 83.16±13.38 | — | — |
| Education level | Secondary nursing school | 78.89±11.17 | 1.29 | 0.277 |
|  | Junior college | 86.70±13.59 | — | — |
|  | Bachelor’s degree | 85.54±13.16 | — | — |
|  | Master’s degree or above | 85.73±13.27 | — | — |
| Marital status | Single | 85.55±13.69 | 3.99 | **0.008** |
|  | Married | 86.10±12.82 | — | — |
|  | Divorced | 78.05±15.64 | — | — |
|  | Widowed | 67.50±19.09 | — | — |
| Department | Internal medicine | 89.52±13.26 | 7.979 | **<0.001** |
|  | Surgery | 85.97±12.59 | — | — |
|  | Obstetrics & gynecology | 84.13±15.79 | — | — |
|  | Pediatrics | 80.93±13.83 | — | — |
|  | Intensive care unit | 80.84±12.62 | — | — |
|  | Emergency department | 80.26±12.26 | — | — |
|  | Outpatient | 87.20±12.51 | — | — |
|  | Operating room | 84.56±12.49 | — | — |
| Professional title | Nurse | 87.44±12.93 | 2.02 | 0.109 |
|  | Nurse practitioner | 85.26±13.16 | — | — |
|  | Senior nurse practitioner | 85.20±13.76 | — | — |
|  | Associate chief nurse or above | 90.00±11.89 | — | — |
| Years of service | 1-4 years | 87.22±13.11 | 2.96 | **0.031** |
|  | 5-10 years | 86.42±13.07 | — | — |
|  | 11-20 years | 85.11±13.29 | — | — |
|  | >20 years | 83.68±13.41 | — | — |
| Monthly income | ≤4000 CNY | 77.50±18.21 | 1.54 | 0.202 |
|  | 4001-8000 CNY | 86.54±14.22 | — | — |
|  | 8001-12000 CNY | 86.14±12.91 | — | — |
|  | >12000 CNY | 85.28±13.28 | — | — |
| Employment type | Officially employed | 86.82±13.11 | 3.391 | **0.001** |
|  | Contract-based | 84.18±13.28 | — | — |

Note. Values are presented as mean ± standard deviation (SD). Differences in subjective well-being across demographic groups were assessed using independent-samples t-tests or one-way analysis of variance (ANOVA), as appropriate. CNY = Chinese yuan.

Supplementary Table S2. Latent-profile model fit indices

| **Classes** | **Log-likelihood** | **AIC** | **BIC** | **aBIC** | **Entropy** | **P-value（LMR）** | **P-value（BLRT）** |
| --- | --- | --- | --- | --- | --- | --- | --- |
| 1 | -8411.808 | 16843.616 | 16894.400 | 16862.636 | — | — | — |
| 2 | -8076.839 | 16185.678 | 16266.932 | 16216.11 | 0.749 | 0.0192 | <0.001 |
| **3** | **-7796.903** | **15637.807** | **15749.53** | **15679.65** | **0.784** | **<0.001** | **<0.001** |
| 4 | -7736.421 | 15528.843 | 15671.036 | 15582.098 | 0.763 | 0.0530 | <0.001 |
| 5 | -7665.826 | 15399.651 | 15572.315 | 15464.318 | 0.729 | 0.0524 | <0.001 |
| 6 | -7632.548 | 15345.095 | 15548.229 | 15421.174 | 0.752 | 0.0088 | <0.001 |

Note. AIC = Akaike information criterion; BIC = Bayesian information criterion; aBIC = sample-size-adjusted Bayesian information criterion; LMR = Lo–Mendell–Rubin adjusted likelihood ratio test; BLRT = bootstrap likelihood ratio test. A dash indicates that the statistic was not applicable. Lower information-criterion values indicate better relative model fit; entropy reflects classification precision.

Supplementary Table S3. Average posterior profile-membership probabilities

| **Assigned profile** | **Class 1 (%)** | **Class 2 (%)** | **Class 3 (%)** |
| --- | --- | --- | --- |
| Class 1 | 88.5 | 11.4 | 0.1 |
| Class 2 | 8.6 | 90.5 | 1.0 |
| Class 3 | 0 | 4.6 | 95.4 |

Note. Rows represent the most likely assigned profile, and columns give the mean posterior probability of membership in each profile (%). Class 1 = resilient; Class 2 = cautious; Class 3 = independent.

Supplementary Table S4. Hierarchical moderation model fit across seven work-environment dimensions

| **Dimension** | **Step** | **R²** | **Adjusted R²** | **ΔR²** | **ΔF** | **Interaction-block p** |
| --- | --- | --- | --- | --- | --- | --- |
| Career development (CD) | 1 | 0.077 | 0.064 | 0.077 | 5.770 | ＜0.001 |
|  | 2 | 0.235 | 0.223 | 0.157 | 240.064 | ＜0.001 |
|  | 3 | 0.435 | 0.425 | 0.200 | 206.092 | ＜0.001 |
|  | 4 | 0.436 | 0.426 | 0.002 | 1.583 | 0.206 |
| Leadership and management (LM) | 1 | 0.077 | 0.064 | 0.077 | 5.770 | ＜0.001 |
|  | 2 | 0.243 | 0.232 | 0.166 | 255.688 | ＜0.001 |
|  | 3 | 0.444 | 0.434 | 0.200 | 209.915 | ＜0.001 |
|  | 4 | 0.446 | 0.435 | 0.002 | 2.332 | 0.098 |
| Nurse-physician relationships (NPR) | 1 | 0.077 | 0.064 | 0.077 | 5.770 | ＜0.001 |
|  | 2 | 0.273 | 0.262 | 0.196 | 313.994 | ＜0.001 |
|  | 3 | 0.456 | 0.446 | 0.183 | 195.339 | ＜0.001 |
|  | 4 | 0.458 | 0.448 | 0.003 | 2.857 | 0.058 |
| Basic support (BS) | 1 | 0.077 | 0.064 | 0.077 | 5.770 | ＜0.001 |
|  | 2 | 0.308 | 0.297 | 0.230 | 387.958 | ＜0.001 |
|  | 3 | 0.477 | 0.468 | 0.169 | 188.443 | ＜0.001 |
|  | 4 | 0.477 | 0.468 | 0.001 | 0.638 | 0.528 |
| Staffing adequacy (SA) | 1 | 0.077 | 0.064 | 0.077 | 5.770 | ＜0.001 |
|  | 2 | 0.297 | 0.287 | 0.220 | 365.470 | ＜0.001 |
|  | 3 | 0.471 | 0.462 | 0.174 | 191.663 | ＜0.001 |
|  | 4 | 0.472 | 0.463 | <0.001 | 0.120 | 0.887 |
| Recognition climate (RA) | 1 | 0.077 | 0.064 | 0.077 | 5.770 | ＜0.001 |
|  | 2 | 0.275 | 0.264 | 0.198 | 318.353 | ＜0.001 |
|  | 3 | 0.454 | 0.445 | 0.179 | 191.354 | ＜0.001 |
|  | 4 | 0.460 | 0.450 | 0.006 | 6.355 | **0.002** |
| Professional autonomy (PA) | 1 | 0.077 | 0.064 | 0.077 | 5.770 | ＜0.001 |
|  | 2 | 0.234 | 0.222 | 0.156 | 238.237 | ＜0.001 |
|  | 3 | 0.433 | 0.423 | 0.199 | 204.293 | ＜0.001 |
|  | 4 | 0.438 | 0.427 | 0.005 | 5.207 | **0.006** |

Note. Each work-environment dimension was examined in a separate hierarchical regression model. Step 1 included covariates; Step 2 added the focal work-environment dimension; Step 3 added personality-profile dummy variables; and Step 4 added two dimension-by-profile interaction terms. R² = coefficient of determination; adjusted R² = adjusted coefficient of determination; ΔR² = change in R²; ΔF = F statistic for the change in model fit. The Step 4 p value represents the omnibus test of the interaction block. The Bonferroni-adjusted significance threshold for seven models was 0.05/7 = 0.0071.

Supplementary Table S5. Final-model focal coefficients across work-environment dimensions

| **Dimension** | **Predictor** | **B** | **SE** | **β** | **95% CI for B** | **t** |
| --- | --- | --- | --- | --- | --- | --- |
| Career development (CD) | Career development (CD) | 1.373 | 0.200 | 0.327 | 0.981 – 1.764 | 6.879 |
|  | Cautious personality (C1) | -12.554 | 0.666 | -0.474 | -13.862 – -11.247 | -18.838 |
|  | Independent personality (C2) | -12.235 | 1.351 | -0.232 | -14.885 – -9.585 | -9.058 |
|  | CDC1 | -0.392 | 0.238 | -0.068 | -0.859 – 0.076 | -1.643 |
|  | CDC2 | -0.456 | 0.316 | -0.045 | -1.076 – 0.164 | -1.444 |
| Leadership and management (LM) | Leadership and management (LM) | 1.572 | 0.218 | 0.342 | 1.144 – 1.999 | 7.217 |
|  | Cautious personality (C1) | -12.597 | 0.655 | -0.476 | -13.883 – -11.312 | -19.226 |
|  | Independent personality (C2) | -11.720 | 1.308 | -0.222 | -14.287 – -9.154 | -8.959 |
|  | LMC1 | -0.494 | 0.257 | -0.079 | -0.998– 0.009 | -1.926 |
|  | LMC2 | -0.057 | 0.346 | -0.005 | -0.737 – 0.623 | -0.165 |
| Nurse-physician relationships (NPR) | Nurse–physician relationships (NPR) | 1.984 | 0.231 | 0.399 | 1.530 – 2.438 | 8.576 |
|  | Cautious personality (C1) | -11.947 | 0.657 | -0.451 | -13.237 – -10.657 | -18.176 |
|  | Independent personality (C2) | -11.700 | 1.298 | -0.222 | -14.246 – -9.154 | -9.016 |
|  | NPRC1 | -0.640 | 0.276 | -0.093 | -1.182 – -0.098 | -2.315 |
|  | NPRC2 | -0.623 | 0.364 | -0.052 | -1.338 – 0.091 | -1.711 |
| Basic support (BS) | Basic support (BS) | 2.139 | 0.245 | 0.400 | 1.658 – 2.619 | 8.728 |
|  | Cautious personality (C1) | -11.752 | 0.646 | -0.444 | -13.019 – -10.486 | -18.205 |
|  | Independent personality (C2) | -11.293 | 1.296 | -0.214 | -13.835 – -8.751 | -8.716 |
|  | BSC1 | -0.323 | 0.287 | -0.045 | -0.887 – 0.240 | -1.126 |
|  | BSC2 | -0.259 | 0.429 | -0.017 | -1.101 – 0.582 | -0.605 |
| Staffing adequacy (SA) | Staffing adequacy (SA) | 1.847 | 0.227 | 0.350 | 1.401 – 2.294 | 8.120 |
|  | Cautious personality (C1) | -11.969 | 0.643 | -0.452 | -13.232 – -10.706 | -18.600 |
|  | Independent personality (C2) | -12.130 | 1.285 | -0.230 | -14.651 – -9.610 | -9.442 |
|  | SAC1 | -0.048 | 0.274 | -0.007 | -0.586 – 0.490 | -0.176 |
|  | SAC2 | -0.209 | 0.428 | -0.013 | -1.049 – 0.630 | -0.490 |
| Recognition climate (RA) | Recognition climate (RA) | 3.310 | 0.366 | 0.435 | 2.592 – 4.028 | 9.044 |
|  | Cautious personality (C1) | -11.842 | 0.662 | -0.447 | -13.141 – -10.542 | -17.877 |
|  | Independent personality (C2) | -10.029 | 1.334 | -0.190 | -12.647 – -7.412 | -7.519 |
|  | RAC1 | -1.538 | 0.438 | -0.140 | -2.397 – -0.678 | -3.509 |
|  | RAC2 | -0.782 | 0.532 | -0.048 | -1.825 – 0.261 | -1.471 |
| Professional autonomy (PA) | Professional autonomy (PA) | 1.944 | 0.254 | 0.353 | 1.446 – 2.443 | 7.652 |
|  | Cautious personality (C1) | -12.540 | 0.663 | -0.474 | -13.841 – -11.239 | -18.914 |
|  | Independent personality (C2) | -11.149 | 1.352 | -0.211 | -13.801 – -8.497 | -8.247 |
|  | PAC1 | -0.938 | 0.308 | -0.121 | -1.543 – -0.333 | -3.044 |
|  | PAC2 | -0.263 | 0.404 | -0.021 | -1.055 – 0.528 | -0.653 |

Note. B = unstandardized regression coefficient; SE = standard error; β = standardized regression coefficient; CI = confidence interval. Estimates are from Step 4 of each dimension-specific hierarchical regression model; covariate coefficients are omitted for brevity. The resilient profile is the reference category. C1 and C2 identify the cautious and independent profile indicators, respectively; interaction terms combine each work-environment dimension with the corresponding profile indicator. Abbreviations: CD = career development; LM = leadership and management; NPR = nurse–physician relationships; BS = basic support; SA = staffing adequacy; RA = recognition climate; PA = professional autonomy.

Supplementary Table S6. Simple slopes of significant personality-profile interactions

| **Dimension** | **Personality profile** | **Simple slope (B)** | **SE** | **t** | **Unadjusted p** |
| --- | --- | --- | --- | --- | --- |
| Recognition climate | Resilient | 3.310 | 0.366 | 9.044 | <0.001 |
|  | Cautious | 1.773 | 0.246 | 7.193 | <0.001 |
|  | Independent | 2.528 | 0.392 | 6.457 | <0.001 |
| Professional autonomy | Resilient | 1.944 | 0.254 | 7.652 | <0.001 |
|  | Cautious | 1.006 | 0.177 | 5.677 | <0.001 |
|  | Independent | 1.681 | 0.316 | 5.317 | <0.001 |

Note. Simple slopes are unstandardized coefficients (B) from the final moderation models, estimated by changing the reference personality profile while retaining the same model specification. SE = standard error. The p values are unadjusted; interpretation of profile moderation was based on the Bonferroni-adjusted omnibus interaction tests across seven work-environment dimensions (α = 0.0071).
